# The Importance of Prions

**DOI:** 10.1371/journal.ppat.1003090

**Published:** 2013-01-31

**Authors:** Glenn C. Telling

**Affiliations:** Prion Research Center (PRC) and the Department of Microbiology, Immunology and Pathology, Colorado State University, Fort Collins, Colorado, United States of America; Washington University School of Medicine, United States of America

While agent host-range and strain properties convinced early researchers of a viral etiology, the once unorthodox postulate that prion transmission occurs by conformational corruption of host-encoded cellular prion protein (PrP^C^) by a pathogenic isoform (PrP^Sc^) is now widely accepted. Indeed, conformational templating is increasingly understood to be a general mechanism of protein-mediated information transfer and pathogenesis. The high infectivity of prions, their capacity to cause neurodegeneration in genetically tractable animal models, as well as the ability to culture prions in cells, or under cell-free conditions using defined components, provide finely controlled experimental settings in which to elucidate general mechanisms for all diseases involving protein conformational templating, and thus to develop integrated therapeutic approaches.

## The Importance of Prion Transmission Barriers

Prion disease epidemics are frequent and, since they are invariably fatal and incurable, of significant concern for animal and human health. Examples include *kuru*, once the leading cause of death among the Fore people in Papua New Guinea, caused by mortuary feasting; the global bovine spongiform encephalopathy (BSE) epidemic, and its subsequent zoonotic transmission in the form of variant Creutzfeldt Jakob disease (vCJD), caused by prion contamination of cattle and human food, respectively; and repeated examples of large-scale animal prion disease epidemics caused by contaminated animal vaccines. The etiologies of chronic wasting disease (CWD) of deer, elk, and moose, and transmissible mink encephalopathy as well, are less well understood. CWD is of particular concern because it is the only recognized prion disease of wild as well as captive animals. Its unparalleled transmission efficiency complicates strategies for controlling CWD, which continues to emerge in new locations and species.

The parameters controlling intra- and interspecies prion transmission are only partially understood. While primary structure identity between PrP^Sc^ in the inoculum and PrP^C^ in the host favors disease transmission, and indeed is the basis of eliminating prion species barriers in transgenic mouse models [1], prions share the ability to propagate strain information with nucleic-acid-based pathogens, and strain properties exert significant influence on agent host range. Prion strain diversity is well documented for scrapie, BSE, and human prions, and most recently CWD. The influence of strain properties on prion host range is exemplified by the spread of BSE prions to humans as vCJD. While the zoonotic potential of newly described cervid prion strains is currently unclear [2], the presence of CWD prions in deer and elk tissues consumed by humans as well as the continued emergence of novel BSE and scrapie strains raise additional public health concerns.

The existence of heritable strain properties in the absence of an agent-specific nucleic acid initially presented a conundrum until evidence that these biological characteristics were enciphered by different PrP^Sc^ conformations [1]. This notion was refined in the Conformational Selection Model [3], which proposes that strains are composed of a range of PrP^Sc^ conformers, or quasi-species, and that only a subset of PrP^Sc^ conformations is compatible with each PrP primary structure. In circumstances where selection allows for the propagation of thermodynamically related PrP^Sc^ conformations, a diseased host may be capable of propagating mixtures of distinct but unstable strains [2], and in other settings, strains may be induced to change biological properties under selective pressure, at least transiently [4].

## The Importance of Host Cell Factors

The limited number of prion-susceptible PrP^C^-expressing cell lines and the ability to isolate subclones of such cells with variable susceptibilities to prion infection support the notion that unidentified auxiliary cellular factor(s) participate in prion replication. Recent studies characterizing the properties of prion strains that replicate in both the lymphoreticular and central nervous systems (CNS) underscore the involvement of tissue-specific factors. Using transgenic mice, Beringue and co-workers compared the ability of brain and spleen tissues to replicate CWD and BSE prions and found that interspecies transmission showed marked tissue dependence, with lymphoreticular tissue being consistently more permissive than brain [5]. The variability of prion strain properties from tissue to tissue within an infected host raises the possibility that assessments of zoonotic potential based on the properties of CNS-derived strains, rather than prions identified in tissues directly consumed by humans [6]–[8], may give rise to misleading estimates of human species barriers to animal prions.

The refinement of protein misfolding cyclic amplification (PMCA) for propagating infectivity using purified components [9], [10] has been an extremely productive means of defining the role of nonprotein cofactors. Recent studies describe the participation of phosphatidylethanolamine in the formation of mouse prions from recombinant PrP (recPrP) [11], specifically as an integral part of the infectious particle regulating PrP^Sc^ conformation, infectivity, and strain properties [12].

## The Importance of PrP Structure

The fundamental event during prion propagation is physicochemical conversion of predominantly α-helical, monomeric, protease-sensitive, and detergent-soluble PrP^C^ into aggregation-prone, protease-resistant, detergent-insoluble PrP^Sc^ that is rich in β-sheet. Determining the mechanism by which this conformational transformation occurs remains a fundamental challenge, and key to this is an understanding of the high-resolution structures of both PrP isoforms. The three-dimensional structure of bacterially expressed recPrP is well characterized and consists of a largely unstructured amino-terminal region, while residues 126 to 218 in the carboxyl-terminus encompass a structured globular domain comprised of three α-helices interspersed with two short sections forming a β-pleated sheet. In contrast, little is known about the structural details of the infectious conformation. Recent experimental studies using mass spectrometry analysis coupled with hydrogen-deuterium exchange indicate a PrP^Sc^ conformation radically different from PrP^C^ [13], which is at odds with the “β-helical” and “spiral” models, in which PrP^Sc^ retains substantial amounts of native α-helices [14], [15]. Undoubtedly, the capacity to amplify highly infectious prions using recPrP by PMCA [10] will greatly facilitate the isolation of PrP^Sc^ for future structural studies.

Another approach has been to develop immunological reagents capable of distinguishing the PrP^C^ and PrP^Sc^ conformations. Evidence for conformational PrP epitopes remained indirect and controversial until the recent mapping of amino acid residues constituting two discontinuous, conformation-dependent epitopes in the structured globular domain [16]. Interestingly, while these monoclonal antibodies recognize their epitopes in the context of the structured globular domain of PrP^C^, they also react with immunoblotted, PK-treated PrP^Sc^, thereby indicating that denatured PrP^C^ and PK-treated PrP^Sc^ re-nature into a common PrP^C^ fold, which is consistent with previous structural studies [17].

## The Importance of the Prion Mechanism in Other Settings

The participation of prions in diverse biological settings ranging from translation termination in yeast, memory in Aplysia, and antiviral innate immune responses has demonstrated the generality of protein-mediated information transfer [18]. Increasing evidence also links the prion mechanism to proteins involved in the pathogenesis of other common neurodegenerative diseases [19]. In the case of Alzheimer's disease (AD), initial evidence of disease transmission to marmosets was confirmed by several groups in transgenic mouse models of AD using either brain homogenates from AD patients, or synthetic amyloid-β (Aβ peptides), even following peripheral inoculation [18], [19]. While early work in transgenic models suggested acceleration of a preexisting condition by inoculation, as was previously demonstrated for transgenic mouse models of an inherited form of human prion disease [20], that disease is truly transmissible was shown by induction of Aβ deposition following injection of AD brain extracts into animals that otherwise do not develop pathology [21]. Similar prion-like transmission has been demonstrated in various settings for other misfolded proteins involved in human neurodegenerative diseases, including the intracytoplasmic proteins tau, also involved in AD and various neurodegenerative diseases referred to as taopathies, and α-synuclein, the primary constituent of Lewy bodies found in Parkinson's disease [18].

## The Importance of Neurodegeneration

Prions offer significant advantages to address the mechanisms of selective neurodegeneration. First, in contrast to animal models of other human neurodegenerative diseases that may require overexpression of multiple mutant transgenes to incompletely mirror only certain features of pathogenesis, all aspects of human and animal prion diseases are recapitulated following their adaptive transmission to laboratory rodents, including early behavioral changes, profound neurodegeneration, and associated clinical deficits. Second, by transgenic manipulations of PrP genes in mice, it is possible to precisely modify responses to prion infections from a variety of human and animal sources and to model inherited forms of human prion disease [1]. Third, prions exist as different strains with defined and reproducible replication kinetics and neurotropic properties.

Recent studies in which the kinetics of disease onset and neurodegeneration were precisely assessed following a controlled prion infection indicated an uncoupling of infectious and neurotoxic PrP species [22]. In other studies using prion-infected mice in which PrP expression was subjected to tight temporal regulation, Mallucci and colleagues demonstrated a critical window of opportunity in which to reverse synaptic dysfunction, and consequently to rescue degenerating neurons [23]. In subsequent studies, her group showed that accumulation of PrP^Sc^ during prion replication caused sustained deregulated activation of the unfolded protein response (UPR), causing persistent translational repression of global protein synthesis, and concomitant synaptic failure and neuronal loss [24]. Consistent with UPR mediated by eukaryotic translation initiation factor, eIF2, in which the α-subunit was phosphorylated (eIF2α-P), both overexpression of GADD34, a specific eIF2α-P phosphatase, and reduction of PrP^Sc^ levels reduced eIF2α-P levels, restored appropriate translation rates, rescued synaptic deficits and neuronal loss, and significantly increased survival. In contrast, salubrinal, an inhibitor of eIF2α-P dephosphorylation, increased eIF2α-P levels, exacerbated neurotoxicity, and significantly reduced survival in prion-diseased mice. The authors point out that UPR activation and/or increased eIF2α-P levels are seen not only in prion disorders but also in patients with AD and Parkinson's disease and suggest that translational control may represent a common pathway for therapeutic intervention to prevent synaptic failure and neuronal loss across the spectrum of disorders involving protein misfolding.
